# Telomeric aging: mitotic clock or stress indicator?

**DOI:** 10.3389/fgene.2015.00082

**Published:** 2015-03-16

**Authors:** Alexander K. Koliada, Dmitry S. Krasnenkov, Alexander M. Vaiserman

**Affiliations:** D.F. Chebotarev Institute of Gerontology, National Academy of Medical Sciences of UkraineKiev, Ukraine

**Keywords:** age-dependent telomere shortening, telomerase, replicative senescence, oxidative stress

Telomeres are regions of tandem arrays of TTAGGG repeats and associated proteins located at chromosomal ends that allow cells to distinguish chromosome ends from double-strand breaks and protect chromosomes from end-to-end fusion, recombination, and degradation (Houben et al., 2008). Telomeres are not linear structures, telomeric DNA is maintained in a loop structure due to many key proteins. This structure serves to protect the ends of chromosomes (Neidle and Parkinson, 2003). Telomeres are subjected to shortening at each cycle of cell division due to incomplete synthesis of the lagging strand during DNA replication owing to the inability of DNA polymerase to completely replicate the ends of chromosome DNA (“end-replication problem”) (Muraki et al., 2012). Therefore, they assume to limit the number of cell cycles and act as a “mitotic clock” (Olovnikov, 1996). Shortened telomeres cause decreased proliferative potential, thus triggering senescence (Blackburn et al., 2006).

Telomere length (TL) is highly heterogeneous in somatic cells, but generally decreases with age in proliferating tissues thereby constituting a barrier to tumorigenesis but also contributing to age-related loss of stem cells. Repair of critically short (“uncapped”) telomeres by telomerase enzyme, which elongates chromosomal ends by synthesizing new telomeric repeats, is limited in somatic cells, and cellular senescence, apoptosis and/or a permanent cell cycle arrest in G1 phase are triggered by a critical accumulation of uncapped telomeres (D'Souza et al., 2013). Telomerase maintains TL by adding telomeric DNA repeats to chromosome ends in prenatal tissues, gametes, stem cells, and cancerous cells. In proliferative somatic cells, it is usually inactive or expressed at levels that are not high enough to maintain the stable TL (Hiyama and Hiyama, 2007). Alternative lengthening of telomeres (ALT) pathway is a recombination-mediated process, which can increase telomere length by thousands of base-pairs within a few somatic cell cycles (Liu et al., 2007). Telomere-initiated cellular senescence is a mechanism of eliminating cells with damaged DNA and protection against cancerogenesis (through the activation mechanisms of cell cycle arrest or double-strand breaks-induced apoptosis), but it may also impair the cell function contributing to degenerative organ failure and organismal aging (Chen et al., 2007; Campisi, 2013). Cellular senescence (a state of irreversible cell cycle arrest) occurring in response to a telomere shortening may be regulated by immune surveillance. In this process, senescent cells interact with their environment by secreting various growth factors and cytokines, being thereby recognized and eliminated by an antigen-specific immune response (Hoenicke and Zender, 2012; Sagiv and Krizhanovsky, 2013). Shortened telomeres have also been observed in a variety of chronic degenerative diseases, including type 2 diabetes, cardiovascular disease, osteoporosis, and cancer (Armanios, 2013; Babizhayev et al., 2014). The specific molecular mechanism by which short telomeres trigger the development of diseases is, however, not yet determined. It has been proposed that telomere shortening *per-se* might not be a direct signal for cell cycle arrest, but rather the consequence of telomere loss (Vaziri and Benchimol, 1996). It can promote a pro-inflammatory secretory phenotype, in turn contributing to a variety of age-related diseases.

Replicative attrition, however, is not the only explanation for age-dependent telomere shortening. Some studies demonstrate that this process can be non-replicative and significantly stress-dependent because of the deficiency of a telomere-specific damage repair (von Zglinicki, 2003). Potential stochastic nature of progressive telomere shortening was shown by Levy et al. (1992). Accumulating research findings suggest that psychophysiological stress may accelerate the telomere attrition from early development, and maybe even prenatally, with potential impact for life-long health (Shalev et al., 2013a; Monaghan, 2014). For example, it has been shown that the more violence children had experienced the shorter were their telomeres from 5 to 10 years of age (Shalev et al., 2013b). In the study by Entringer et al. (2011), young adults whose mothers were exposed to severe stressors during pregnancy (for example, due to the death of a close family member) had more short telomeres than those subjects whose mothers had a relatively stress-free pregnancy. Several studies also indicate that effects of exposure to stress early in life may persist into adulthood. In research by Surtees et al. (2011), it has been demonstrated that the more adversity women had experienced during their childhood the shorter were their telomeres in adult life. By investigating the hypothesis that psychophysiological stress may impact health by modulating the rate of cellular aging, Epel et al. (2004) examined the TLs and telomerase activity levels in mothers of healthy or chronically sick children. The second group was predicted to be more chronically stressed than the first one. In this research, mothers of ill children demonstrated substantially reduced telomerase activity which was equivalent to the 9–17 years of additional cellular aging as compared to those women who had healthy children, and rates of cellular aging were obviously associated with levels of stress exposure. Substantial telomere attrition was also revealed among caregivers of AD (Alzheimer's disease) patients. In this occupational group, telomere shortening was clearly associated with symptoms of depression, in comparison to control subjects matched for gender and age (Damjanovic et al., 2007). The correlation between level of perceived stress and TL was clearly confirmed in a recent meta-analysis conducted by Schutte and Malouff (2014).

Oxidative stress is one of the most important stress factors causing telomere shortening (Houben et al., 2008; Zhang et al., 2014). Telomeric DNA is known to be more susceptible to oxidative damage than non-telomeric DNA, owing to the high content of guanine residues and a reduced DNA repair capacity in telomeric regions (Gomes et al., 2010). Triple guanine repeats of telomeric DNA were shown to be particularly prone to oxidative damage, and they may generate oxidatively modified bases such as 8-oxoguanine which is the main substrate for the base excision repair (BER) pathway (Kawanishi and Oikawa, 2004). Wang et al. (2010) demonstrated that oxidative damage of guanine plays an important role in disruption of telomere integrity and presumes that BER pathway is substantially involved in repair of oxidative base lesions in mammalian telomeres. Moreover, telomeric DNA, in contrast to genomic DNA, is deficient in repair of single strand breaks and double strand breaks, and telomere dysfunction is believed to mediate the effect of oxidative base damage to abnormal nuclear morphology (Coluzzi et al., 2014). In human cell lines, telomeres generally shorten by 30–200 base pairs at each round of DNA replication, but only approximately 10 base pairs of this reduction are a consequence of the end-replication problem; the remaining loss is likely owing to oxidative damage (Monaghan and Haussmann, 2006). Systemic chronic inflammation to accelerate aging via ROS-mediated telomere dysfunction and cell senescence was shown in mice (Jurk et al., 2014).

In several studies, it was found, that DNA damaging agents cause telomere erosion, primarily, by high levels of reactive oxygen species (ROS) regardless of age of the cell donor. For example, in the Richter and von Zglinicki (2007) study, high levels of oxidative stress cause accelerated rates of telomere erosion and, ultimately, arrest of the cell cycle in cell cultures derived from donors of various ages (embryonic to adult). Moreover, it was revealed that over-expression of superoxide dismutase in a cell line with low antioxidant capacity slowed erosion of telomeres (Serra et al., 2003). Further evidence for non-replicative cause of telomere shortening comes from the fact that the telomere erosion rate varies in a stochastic manner among cells in fibroblast clones, derived from single cells, thereby causing sub-clonal heterogeneity in replicative lifespan (Martin-Ruiz et al., 2004). Recently, it was found that such cell-to-cell heterogeneity may come from stochastic heterogeneity in oxidative DNA damage caused, e.g., by variations in the cell's metabolic processes (Snijder and Pelkmans, 2011; Trusina, 2014). Another pathway for non-replicative telomere erosion relates to non-canonical function of telomerase, mainly, protection of mitochondria against oxidative stress (Saretzki, 2014). The fact that TERT is transported out of the nucleus under long-term low level oxidative stress suggests that protection of mitochondrial DNA might have higher priority than protection of telomeres maintenance under stress (Ahmed et al., 2008).

The complexity of processes underlying age-related telomere erosion came from several longitudinal studies of telomere dynamics *in vivo*. Research of TLs in blood cells of snakes, birds, and humans unexpectedly revealed, that no TLs changes with aging were found (for review, see Eisenberg, 2011). Moreover, the faster age-related decline in leukocyte TLs was observed among subjects with longer telomeres at baseline early-adult examinations, suggesting that longer telomeres provide bigger amount of targets for oxidative stress-related telomere erosion (Aviv et al., 2009). Traditionally, it was assumed that telomeres are stable structures, which may be changed only in unidirectional way—shortening over the lifetime. Today, however, it has become increasingly clear that telomeres shortening over time in an oscillatory rather than linear fashion and they may be either shortened or lengthened under certain conditions (Epel, 2012). Several pilot studies indicate that treatment procedures targeting to reduce stress, e.g., meditation, along with the enhanced physical activity and changes in dietary patterns, can slow or even reverse telomere shortening owing to the elevated telomerase activity (Jacobs et al., 2011; Blackburn and Epel, 2012). The elongation of telomeres may be caused by the telomerase-mediated extension or appear due to the “pseudo-telomeric lengthening.” The latest is due to the fact that, since TLs are commonly measured in a mixed leukocyte population, mean TL can increase because of a redistribution of cell subpopulations, i.e., change in the percentage of various cell types in the blood samples.

Consistent evidence suggest that telomere erosion may be a common mechanism that triggers all major processes underlying aging, including mutation accumulation, mitochondrial dysfunction and stem cell failure (Armanios, 2013), as well as epigenetic changes likely through silencing of gene expression by formation of long-range chromatin interactions (Fojtová and Fajkus, 2014; Misteli, 2014). Induction of senescence occurs in reaction to a critically short telomere length and to the triggering of DNA damage response. Therefore, unraveling the factors causing telomere attrition may be important for further progress in understanding the basic mechanisms of aging. In this context, it is particularly important to establish the relative significance of end-replication and oxidative damage events in the age-related telomere erosion.

Given data from recent studies, a concept that replicative senescence is a “clocked” and stepwise process seems doubtful, and repeatedly reported reproducibility of both replicative lifespans and rates of telomere shortening could be the result of stochastic rather than programmed events (von Zglinicki, 2003). In other words, it seems that telomeres can be an indicator of stress-induced damage level rather than a mitosis “counter.” Moreover, considering the fact that oxidative stress represents a common causative mechanism for both age-related telomere shortening (Houben et al., 2008) and age-associated disease (Richardson and Schadt, 2014), there are reasons to believe that relationships between TL and morbidity or mortality are non-causal, and TL can be an indicator of previous exposure to oxidative stress that may, in turn, cause both greater telomere shortening and higher risk of chronic disease. Thereby, perceived stressful events, though correlated with TL, may likely have independent effects on health and longevity (Eisenberg, 2011). By summarizing recent research findings, Trusina (2014) conclude that recently obtained knowledge “shift the telomere paradigm from a simple clock counting cell divisions to a more complex process recording the history of stress exposure within a cell lineage.” This point of view, based on the accumulated evidence, appears plausible, and requires further investigation.

## Conflict of interest statement

The authors declare that the research was conducted in the absence of any commercial or financial relationships that could be construed as a potential conflict of interest.
